# Foreign

**Published:** 1841-05-08

**Authors:** 


### FOREIGN.

*Observations on Vaccination and Small Pox, more especially with reference to the theory of Vaccine Influence, and the relation subsisting between the Cicatrix and the character of the Consecutive Variola.* By George Gregory, M. D., Physician to the Small Pox Hospital.—The observations of the author in the present paper are intended to point out, first, the alarming increase of small pox in the metropolis, as shown by the books of the Small Pox Hospital; and, secondly, the insufficiency of the appearance of the cicatrix of the former vaccination as a test of the degree of protection afforded by the process.

Upon the first point he adduces the fact, that the admissions in the first three quarters of 1840 only amounted to one hundred and forty-two, being at the rate of sixteen per mensem, while in the twenty-five days immediately preceding the reading of the paper, they amounted to ninety-three, being at the rate of nearly four per diem, a greater number than was ever admitted in one month since the establishment of the hospital in 1746. Of three hundred and sixteen cases admitted in 1840, one hundred and ninety-four had not been vaccinated, of whom eighty-seven died, or forty-five per cent! one hundred and twenty had been vaccinated, of whom only eight died, being at the rate of seven per cent.; the remaining two had had the small pox previously. Of the three hundred and sixteen patients, forty-seven were under five years of age, of whom twenty-eight died; forty-five between five and fifteen, of whom nine died; two hundred and twenty-four were adults, of whom fifty-eight died. The total mortality was ninety-five, or thirty per cent. on the gross admissions. With reference to the second point, the author entered at some length into an explanation of the causes by which the many observed varieties in the appearance of the cicatrix may be explained; and presented the society with two series of well-marked cases, in the first of which severe small pox occurred in cases presenting perfectly normal cicatrices; whilst, in the second, the opposite anomaly presented itself, the lightest and truly varicelloid eruptions co-existing with small and very imperfect cicatrices. In the conclusion of this paper, the author expresses a doubt of the conclusion seemingly derived from the late experiments of Mr. Ceeley, of Aylesbury, of the identity of the vaccine and variolous poisons.

Various questions were put to Dr. Gregory after the reading of his very interesting paper, having reference to vaccination and variola. The more interesting of the points commented upon were, first, the question of the severity of the present epidemic; secondly, the proper period of performing vaccination; thirdly, the ages at which patients affected with small pox after vaccination were vaccinated; fourthly, re-vaccination; and, fifthly, the new lymph, or variola-vaccina.

As to the first, Dr. Webster contended that small pox was not at the present time so general as Dr. Gregory's paper would lead us to believe. He compared the mortality of 1838, the year of the last epidemic, with that of the year 1840, and showed by the tables of the registrar-general, that the mortality in the former was two-thirds more than the mortality of the latter year.

Dr. Gregory explained that the present epidemic did not commence until October, 1840; that of 1838 commenced in the same month, 1837; Dr. Webster would not arrive at a proper conclusion unless he limited his calculations to the last three months of 1840, and compared the deaths occurring in them with those taking place in the same months of 1837. It was known that epidemics required six months to reach their height, and as long a period to recede; that of 1837 reached its height in May, 1838; he expected the present would be most prevalent in May or June of the present year. Under all circumstances, he believed that the present epidemic was not so general as that of 1838. The Small Pox Hospital's entries and deaths had been found during the last thirty years always to bear a direct relation to the number of cases of, and death from, small pox throughout London, and he considered that they afforded a sufficient field on which to found a statistical inquiry. On the second point, Dr. Gregory observed, that so long as the vaccine vesicle had all the usual characteristics, and there was sufficient constitutional disturbance, it mattered little as to when vaccination was performed, whether in the first month, or the first year of life. The difficulty of vaccinating very young children did not depend upon want of susceptibility in the patient, but on the absence of that plumpness which was necessary to be present for the proper performance of vaccination. About the fourth month, this plumpness being usually present, was an excellent time to perform the operation.

Mr. Ceeley agreed with the statements of Dr. Gregory, but believed that vaccination might be properly performed in children at the very earliest age, if the skin were merely scratched with the lancet, instead of an attempt being made to insert the lymph by means of puncturing the skin. To answer the third question there was much difficulty; all the information that could be gained from patients being merely, that they were vaccinated in early life. Dr. Gregory had noticed, however, that more cases of small pox had followed vaccination when performed at the adult period, than when it had been effected in infancy. He thought this showed that infancy was the proper period for vaccination to be performed in, and that the effects of the operation were more decided at this period of life than at the adult period, because in the former a less mass of fluid was required to be impregnated with the protective influence of the virus. The fourth month being a time when the constitution was not affected by dentition, or other contending influences, was the best period to vaccinate. As to re-vaccination, he had little experience in the matter; but this he had observed in a family re-vaccinated under his own eye,—that neither the age of the patient, nor the appearance of the cicatrix upon the arm, appeared to have had any effect upon the vaccination; in some there was a mere papular eruption; in others, the vesicle went through its usual course. On the fifth point, Dr. Gregory made inquiry of members whether or no they had used the new lymph—the variola-vaccine, and if so, with what results? Was it in any way different from the lymph which had been in constant use from the time of Jenner to the present? For his own part he believed it was less certain in its results, and in this respect bore a great analogy to the small pox itself. He had found, for instance, that there was no certainty in the kind of vesicle it would produce; in one person it would be mild, in another irritable; as in small pox, we found the confluent sometimes produced mere varioloid disease, and mere varioloid disease sometimes the most malignant kind of small pox. Mr. Ceeley believed that there was no material difference in the two kinds of lymph; the varieties observed in its effects being dependent upon the soil into which it was inserted, and the season in which it was used.—*Trans. Roy. Med. and Chirurg. Soc., in Prov. Med. and Surg. Journ.*

The great prevalence of small-pox in Philadelphia at the present moment, induces us to insert, as highly interesting to our city readers, the following

*Reflections on the treatment of Variola, by the "ectrotic method," principally with a view of preventing the formation of Cicatrices.*

[Extracted from a paper read before the Parisian Medical Society, by J. F. Olliffe, M. D., President of the Council.]

It is not my intention to enter into an historical account of what has been written on the origin and progress of variola, nor to follow the disease in its different migrations and wanderings; still less do I purpose laying before you the innumerable modes of treatment which, at different periods, have been employed against it. It would be an ungrateful and fastidious task to wade through the mass of matter which has appeared on the subject; the only effect thus produced would be to confuse our ideas, and to convince us that, until very lately, no rational treatment had been proposed to combat the ravages of one of the most dreadful scourges that ever afflicted mankind.

Small-pox is not confined to one climate, nor to any particular class of persons. All countries, all ranks, individuals of all ages, fall a prey to its baneful influence; whilst the hideous scars which frequently deform those who resist its fatal effects, render them objects of pity and commiseration.

In my remarks this evening, I shall endeavour to prove that an external topical treatment, suitably directed, is the *only rational* one, in cases of variola; and I hope to convince those of my auditors, who are not as yet very conversant with the new French doctrines on the subject, of the efficacy of such treatment. I shall dwell on those therapeutical agents which appear to me to have a superior power in subduing the disease, *by preventing the formation of pustules*, and, consequently, of cicatrices; whilst I shall glance at, without stopping to discuss their merits or demerits, several recent suggestions as to the treatment of variola, which have been thrown out by authors both in France and elsewhere.

In the treatment of all varieties of small-pox, whether of the discreet, semi-confluent, or confluent sorts, two curative methods are to be observed; the one may be called, and in fact has been termed *indirect*, the other, direct. The former is purely antiphlogistic, and embraces the general range, depletion, low diet, &c. The latter directly grapples with the exanthem by topical action, and to its employment your attention shall be particularly directed. As to the prophylactic measures, represented by inoculation, vaccination, &c., considerations on this interesting head do not come within the compass of the present paper.

The etiology of the disease must, likewise, be considered as foreign to my subject. Were

I to discuss the nature and causes of variola, I should not thereby throw much light on the *rationale* of the treatment, which alone is to be the object of my research; for the causes are still wrapped in mystery, as the numberless hypotheses of those authors who have written on them abundantly prove. Besides, were I now to moot this question, even in a very superficial manner, I should swell this paper into a volume. Those, however, who wish to glean valuable ideas on the etiology of small-pox, may consult Velpeau's article in the "Revue Medicale," of 1825, and M. Gendrin's "Historie Anatomique des Inflammations," vol. i. p. 398, *et seq.*

Before we enter on the study of the several topical methods which have been proposed, and before we discuss their advantages or inconvenience, let us establish the three following propositions, the truth of which we shall admit *a priori*, reserving to ourselves to prove their accuracy, by deductions drawn from our arguments at the close of the essay.

Prop. 1. I admit, with M. Velpeau and others, that variola is a specific affection, having its *principal* seat in the follicles of the dermis. It is quite unnecessary for me here to describe the different sorts of variola, its duration, symptoms, and complications.

Prop. 2. The principal indication in the treatment of *incipient* variola is to check the progress of the eruption, and to prevent the formation of pustules; each pustule ought to be considered as a nucleus of inflammation.

Prop. 3. Resorption, in cases of variola, far from being attended with accidents, is, on the contrary, always followed by beneficial results.

If we admit these propositions, (and I repeat, that what follows will, I trust, demonstrate them,) it is evident that any topical treatment, having for its object to counteract the eruptive process, will at the same time exert a generally salutary effect. In consulting most of the ancient writers on small-pox, we seek in vain for any therapeutical method directed against the eruption itself; they supposed that it was dangerous to suppress or modify it, and they advised that all efforts should be directed towards obtaining a plentiful *crop* of pustules; it is much to be regretted that the great majority of practitioners of the present day entertain the same erroneous and absurd opinion, and that many inculcate it both in their lectures and writings, thus endangering human life, and favouring the production of deformity. I trust to be able to prove to you, that the precept laid down by Cotugno, and lately reproduced and supported by M. Roche, is fallacious in the extreme. Cotugno says, "*Morborum curatio ita fere instituenda est in variolis, uti instituere-tur, si variolæ non adessent.*"

A modern writer, whose recent work on general pathology just now enjoys a considerable share of public favour, has emitted a preposterous doctrine, which, I hope, for the benefit of suffering humanity, will find few followers. "Les affections varioleuses ne peuvent pas être guéries; on les surveille, et voilâ tout, mais ce serait folie que de chercher à les guérir" (!!!)—Dubois "Pathologie Générale," vol. ii., p. 547.

Let us now rapidly pass in review the different topical remedies employed at various periods in variola.

Previously to Sydenham, the stimulant system was in vogue: heat, cordials, spices, and other such remedies, were had recourse to, and but too frequently proved to be the means of exasperating the malady, and of endangering life. Sydenham destroyed this barbarous treatment, and established as a principle, that with a view of moderating and keeping under the fever, fresh air, cold water, acid drinks, and purgatives, should be employed; to these were added, according to the circumstances and the stage of the disease, blood-letting, blisters applied to the extremities, cold baths, narcotics; but it never occurred, even to the master mind of Sydenham, that the grand secret was to arrest the progress of the eruption, or to modify its nature.

Cotugno was the first to direct attention to the means of preventing the disfiguration of the face. He confined himself to recommending that it should be frequently bathed with tepid milk and water, arguing that a state of continual moisture would be efficacious in opposing the complete developement of the pustules. This work, "De Sedibus Variolorum Syntagma," was published at Vienna in 1771; and from that period down to the year 1825, not a single suggestion worth attending to, was made respecting any external treatment.

In 1825, the researches of Messrs. Bretonneau and Serres were published in the "Archives de Medecine;" they advocated the employment of cauterisation in variola, and gave the name of "*methode ectrotique*" to their discovery; they were joined by Monsieur Velpeau, and these three eminent practitioners agreed as to the *principle* of their curative treatment; but as their mode of operating somewhat differed in detail, I may, perhaps, be allowed briefly to allude to each. M. Bretonneau pierced the summit of each pustule with a gold or silver needle, charged with a small portion of lunar caustic in powder; he cauterised on the second, or at latest on the third day of the eruption, with the double intention of procuring the abortion of the pustules, and preventing the formation of scars; M. Guersent, physician of the Hôpital des Enfans Malades, repeated M. Bretonneau's experiments.

To M. Serres belongs the credit of creating the *methode ectrotique*, properly so called. The word ectrotique is derived from the Greek

εκτρωσις, or εκτρωτικος, which means "procuring abortion;" and I cannot see why it should continue exclusively to be applied to M. Seres' system, which, as we shall presently see, is not the most eligible one. M. Serres proceeded, in confluent small-pox, by what he called "cautérisation en masse." He passed over the eruption a pencil dipped in a solution of nitrate of silver, the proportion from fifteen to forty-five grains per ounce, of distilled water; in the distinct or discreet variety, he was not accustomed to open the insulated pustules, whilst M. Bretonneau invariably did so, but he touched each externally with the caustic. M. Serres affirmed that this topical application, besides causing the abortion of the pustules, opposed the occurrence of phrenitis, otitis, and ophthalmia, which so often appear in the course of the disease which now occupies our attention.

M. Velpeau completely removed the apex of the pustule with the point of a lancet, and then introduced into its cavity the sharpened end of a pencil of nitrate of silver.

Such is a succinct *résumé* of the "méthode ectrotique." Those who wish for a detailed account of the experiments, may consult the "Archives de Médicine," of June 1825, and the "Revue Médicale" for 1825, p. 516, *et seq.*, as also the samè Review, 4th vol., 1828. The inventors of this method have summed up their ideas in the following conclusions:

If the cauterisation be performed on the second day of the eruption, or at *latest*, on the *third*, no pustules are formed; if on the fourth day, the result is uncertain; if it be delayed until the fifth or sixth day, the progress of the eruption is not modified, and cicatrices remain.

The ectrotic method, when it first came into practice, caused a cousiderable sensation in Paris; all the physicians of the metropolis gave it a trial; but it is now completely obsolete; and I perfectly agree with M. Rayer, M. Chomel, and other high authorities, that it should be so, and for the following reasons: 1st, its exhibition is attended with much pain, and, as M. Guersent justly remarks, (Dictionnaire de Med. Art. Variola, in 25 vol.) is it not a serious question whether the excessive pain accompanying the cauterisation, and the inflammatory reaction which ensues, do not tend to provoke those cerebral affections which, M. Serres affirms, are thereby obviated? In case, however, the méthode ectrotique be employed, M. Serres' plan, that of the *cautérisation en masse*, is preferable to the others; it is less painful, and the two others are, in most cases, impracticable. How is it possible to cauterise each pustule in a case of confluent variola? Few individuals would submit to have all their pustules opened, one by one; and fewer physicians would have patience to get through so tedious an operation.

Before I take leave of the méthode ectrotique, I am bound to say, that although it is now almost obsolete, still its momentary and fleeting existence has been productive of immense service to humanity. Its invention will constitute an era in the history of the treatment of small-pox; for its authors have directed the attention of the medical world to a new and effectual mode of combating the progress of the disease: already are the fruits, of which they have sown the seeds, ripening, and a salutary and rich harvest of facts will, I am confident, be, ere long, appended to the annals of therapeutics. But I must not anticipate; let us pursue our cursory review.

M. Eichhorn recommends the employment of vaccination, when the eruption commences. He loses no time in making a number of incisions, (forty or fifty,) into each of which he introduces a quantity of vaccine matter; he assures us that if this practice be persevered in, no case of variola, however virulent, will be fatal. M. Rayer (see Dictionary of Med., in 15 vol., article Variole,) has tried M. Eichhorn's treatment on two patients, both of whom died; but he announced, in 1835, his intention of recurring to his experiments. I know not if he have done so.

The puncture of the pustules, and the careful expression of the purulent matter contained in them, has been tried, and, in some cases, attended with signal success; this system, though of recent adoption in our countries, is of very ancient date. It owes its origin to the Arabs; it has been repeated in France, by Messrs. Rayer and Bonnet; in England, by Dr. Stewart; and in Germany, by Hufeland. But the difficulties which its execution offers are analogous to those we have noticed in alluding to the *méthode ectrotique*, and must ever form a formidable, if not an insuperable, barrier to its adoption.

As to the effects of the refrigerant system, such as exposure to cold air, the employment of cold baths, &c., I am not aware of a single case of variola being on record, in which these remedies have modified the course of the eruption.

Dr. Picton, of New Orleans, published, in 1832, in the American "Journal of Medical Sciences," a series of cases of variola, which had been treated by him in the general hospital of the above named city; his sole therapeutic agent was the absence of light. He affirms that all his patients were cured, *and that not one was pitted.* I own that I am somewhat sceptical as to the authenticity of results so extraordinary.

At a late assembly of the Academy of Medicine of Paris, M. Legrand read an interesting paper, on the employment of gold leaf in small-pox. This practitioner applies to the face of the patient a coating of a solution of gum Arabic, over which he lays the metallic leaf, thus *gilding* the visage. M. Legrand's Memoire, of which an analysis was given in the Archives of August, 1839, was favourably received by the academy.

From what precedes, must we not acknowledge, gentlemen, that we have not hitherto met with any indication, or any basis, whereon we may found a rational therapeutic construction? But I now hasten to perform an agreeable duty, to advert to another topical treatment; one teeming with importance, and which will, I am convinced, operate a total revolution in the curative measures henceforth to be opposed to the ravages of small-pox. I allude to the external use of mercurial preparations—and here, allow me a short retrospective view, permit me briefly to endeavour to trace the history of the employment of mercury in variola.

Mercury, used internally, has been administered at all periods. It was now and again advocated, and as often abandoned; but it seldom, if ever, entered into the minds of physicians to employ it externally. It was frequently remarked that variola assumed a mild aspect when it attacked individuals who had undergone a course of salivation by mercury; and the following passage, which I extract from the erudite work of Mason Good, (vol. iii., p. 110,) will serve to elucidate what I here advance, namely, that mercury, previously to the researches which I shall presently notice, seriously occupied the attention of medical men:

"Mercury appears to have a specific influence on the action of variolous matter; perhaps, as in the case of syphilis, upon the quality of the matter itself; for though, when considerably diluted with water, it is still capable of propagating the disease by inoculation; yet Von Wensel has shown satisfactorily that when triturated with calomel it loses its energy, and in inoculation becomes inert and useless. Mercury has since been denominated in Germany the *remedium pancreston*, and has certainly supported its character as the best corrector of small-pox we are acquainted with, from a period antecedent to the introduction of inoculation into Europe, to the present day." "Physicians who attend hospitals," says Sir George Baker, "have frequently observed the small-pox to be particularly mild in those patients who have happened to receive the infection soon after a mercurial ptyalism; and inoculation is said to have been a much more successful practice in some of our American colonies, since the use of calomel has been there introduced into the preparative regimen." *

Malouin, in his "Chimie Médicinale," relates the case of a woman who was under treatment for a syphilitic affection; she had at the same time a mercurial plaster on the sacrum, over a tumour situated in that region. This plaster had been applied for some days when she was attacked with small-pox; she then removed it. A violent pustulous eruption took place, which covered the whole body, except the part which had previously been in contact with the plaster. This case deserves to be noted, for it became the *point de départ*, the starting post, of a series of admirable experiments. Rosen de Rosenberg, a German physician; Zimmerman, Sulger, and others, employed the mercurial plaster; I have already mentioned the opinions of Von Wensel.

Those insulated facts were lost to science, when M. Serres commenced his researches, which have been continued by several others, and which have lately, in the hands of M. Briquet, been followed by the most valuable results. The first, or, at least, the principal notice of these experiments, was published in the "Archives de Medicine," vol. xxx., 1825, by M. Gariel, interne at the Salpetrière. This article contains the statement of eight cases of variola, six semi-confluent, and two confluent, all treated and cured by mercury. The first patient was a young man, aged twenty-five years. He entered the hospital with an abundant eruption. The plaster of *Vigo cum mercurio** was applied to the left arm, and on the right was placed a common diachylon plaster. Under the mercurial plaster *no pustules appeared;* whilst on the right arm the progress of the eruption was unmodified. No mercurial topic was applied to the face of this patient, as M. Serres dreaded the occurrence of cerebral symptoms; *deep cicatrices* remained after the recovery of the patient.

The second case occurred in February, 1825. I mention this date with precision, as the patient named Bouret, ætat. fifteen, was the first who was treated by the general topical system. His face was covered with the plaster; a part of which he tore off during the night which followed its application. *The denuded surface was the seat of suppurating pustules, whilst on that portion of the visage which continued subjacent to the plaster their abortion was effected!*

The last case narrated by M. Serres, is that of a man named Odard. He had not been vaccinated. He was affected with violent confluent variola; the pimples were small, scarcely raised above the level of the epidermis, and

* The use, or rather abuse, of calomel in America, is carried to an incredible degree. An eminent American practitioner, at present resident in Paris, told me, a short time since, that he had taken one hundred and forty grains in twenty-four hours!

* As the plaster of Vigo will be frequently mentioned in the remainder of this paper, I here give its composition, according to the French pharmacopœia.

| | |
|---|---|
| Mercure - - | 95 parties. |
| Styrax liquide - | 48 |
| Emplatre simple - | 312 |
| Cire, resine, terebinthine, aa. 16. | |
| Gum ammoniac, bdelluin, encens et myrrh, aa. 5. | |
| Safran - - - | 3 parties. |
| Esprit de lavande - | 2 |

surrounded with a brilliant red areola. The Vigo plaster was applied, and allowed to remain seven days; on its removal, it was found that no suppuration had been established, with the exception of four pustules, and these were situated near the mouth, and had not been in contact with the plaster. This patient was radically and rapidly cured, and no scars were manifested. I must not omit to state, that the comparative effects of other topical remedies were tested, but in no case did they in any way modify the eruption.

From the foregoing results, M. Serres drew the following inferences:

1. The plaster of Vigo cum mercurio produces a complete abortion of variolous pustules.

2. Diachylon, porphyrized charcoal, gum arabic in solution, and all other topics hitherto applied, (with the exception of litharge,) with the intention of causing this abortion, have completely failed.

3. The abortion takes place, not only when the pustules are yet in a state of papulæ, but even when they are in full suppuration. (Here I must differ from M. Serres, for M. Briquet's researches prove that the appearance of the pustules in the suppurating state renders the effect of the mercury altogether void.)

4. The abortion takes place by a process totally different from that which is effected in *varioloid* affections. In the latter it is to be attributed to the prompt desiccation of the *vesicle*, whilst in variola the pimple does not open at all; no pus appears, no scales are formed; resorption is effected, and, consequently, no scars can possibly occur.

5, and lastly. The greater part of the body should be covered with the mercurial plaster.

In 1837, M. Gariel defended an inaugural thesis on the subject before us, at the Parisian Faculty of Medicine; but little attention was paid to his remarks, or to those of M. Serres. Even M. Rayer, in his last edition of the "Maladies de la Peau," alludes to mercurials in variola in a very cursory manner, and professes his ignorance of their mode of action. Things were in this state when, in October, 1837, a small-pox epidemic broke out in Paris and elsewhere. M. Briquet, of the Hôpital Cochin, was thus afforded an opportunity of giving mercury a fair trial; and the remarkable consequences of his sagacious and well-directed experiments are such as to establish, in my opinion, a solid, rational, and SPECIFIC treatment of small-pox, in all its diversified varieties. I regret that the limited space to which I am obliged to confine myself, and the length, I fear unreasonable, to which this paper is being extended, will preclude me from giving aught else than a very concise view of M. Briquet's labours. I shall, however, endeavour to lay before you their most prominent features.

It is well known that the violent symptoms, which constitute the *prodrome* of variolous affections, altogether cease, or are considerably diminished, when the eruption appears. The patients, even those labouring under the most confluent small-pox, remain a day or two in comparative comfort; but this calm is transient and delusive; it is but the precursor of a storm. The accidents re-appear with increased intensity, and continue unabated until the process of suppuration is terminated.

Before we throw an analytical glance over this part of M. Briquet's treatment, let us for a moment contemplate the dreadful state of the unfortunate patient attacked with violent confluent small-pox. Innumerable thronging pustules cover his whole body; his face swells to an immoderate size; the eyelids are ulcerated; the ocular conjunctiva is attacked; and the visual functions are often consecutively destroyed; his features are the seat of a vast ulceration; a hideous scab masks his visage, and causes excruciating torture; it is interrupted here and there by fissures, through which ichorous fœtid fluid escapes; the compression, by this scab, of the subjacent parts, may serve to explain the cerebral symptoms which now manifest themselves: this ichor is peculiarly virulent; its course is marked with trails of vesicular and fiery erythema over different parts of the body; the head is oppressed, the brain delirious; the nose and mouth are, by degrees, stopped up; dyspnœa, then suffocation, occur; the inspirator muscles endeavour, but in vain, by the most convulsive efforts, to dilate the lungs; asphyxia often ensues; or, if it do not, the epidermis suddenly becomes flattened, the features sink, the coma increases, and death closes this scene of horror. Frequently, above all in hot climates, (and I witnessed not long since a case of this sort at Naples,) bubonous ulcers appear in the groins; this fatal complication sometimes occurs in our own countries; we find it thus alluded to by Hucksan:—"Variolæ epidemicæ interdem crudo diffluent ichore, qui subjectam carnem erodit, imo et nonunquam ipsâ gangrenâ afficit." Should all the details of this miserable train of symptoms not co-exist, should a cure be effected, the patient bears, for ever, imprinted on his brow, the pernicious brand of the enemy. And here I would ask, Why should not the prevention of the formation of those frightful scars form a principal indication in the treatment of variola? Are we not daily witnesses of the disastrous consequences of disfiguration of the features to those who are visited by the disease? Do not the prejudices of the world, prejudices which I deplore, but which, unfortunately, nevertheless exist, all but exclude disfigured faces from the pale of society? How many heart rending instances of faded beauty, disappointed love, destruction of long-cherished affections, all consequent on the ravages of variola, daily meet the aggrieved regards of the physician? Is it not, then, praiseworthy in the medical philanthropist to combat *à outrance*, the withering inroads of so horrible a scourge? M. Briquet has, doubtless, gone far towards the attainment of so desirable an end.

His first papers were published in the "Archives" of September and October, 1838, (vol. xxx., pp. 5 and 133.) In the brief notice to which I am compelled to limit my present observations, I shall first expose the new plan of treatment, and then hazard a few remarks as to the mode of action of the remedy.

Mercury, as a topic, ought to be employed in all the varieties of small pox, whether simple, confluent, or modified. Its effect is either to prevent the development of the pustules, or so to modify them that they become mere abortions. The whole face should be covered with a mask of the Vigo plaster, merely leaving a space for the mouth, nostrils, and eyes. A little mercurial ointment is applied to the eyelids.* By the application of these plasters, the exanthem either undergoes entire resolution, or is converted into *vesicles* or tubercles. The resolution is either primitive or secondary. The former takes place when the eruption is still papular; so that the papulæ, which, without the application, would have passed into pustules, disappear completely under its influence. The plaster often, indeed generally, changes the nature of the eruption; by converting it into a vesicular or tubercular form. When it assumes the vesicular form, it differs little in appearance from herpes; the vesicles contain a lymph-like serosity, and are surrounded by a light red areola; and a most remarkable circumstance is, that the skin between the insulated vesicles is pale, and never swollen and red, as it is in small pox. No *suppuration ever takes place.* A day or two after the removal of the plaster the vesicles dry up, without any desquamation; and as for cicatrices, M. Briquet has never seen any form after his treatment. He totally differs from M. Serres respecting the utility of the plaster when the eruption has become pustular. The plaster is allowed to remain for three days in simple small pox, and for four in confluent. M. Briquet tried, as did M. Serres, the effects of pressure, of lead plasters, and a host of other topical remedies; but always without the effect of modifying, in any way, the eruption. Thirty observations, some of them relative to cases of the most grievous kind, attest the unparalleled success of M. Briquet's experiments.

* Since I wrote the above, I have received a communication from my respected friend, Dr. Podaliri, of Rome, who informs me that he has been always in the habit of applying mercurial ointment to the eyelids, and round the lips of his variolic patients, and that he has universally remarked the *total absence of pustules* on the parts covered by the ointment. Had Dr. Podaliri, in the course of his extensive practice, *generalised* this method, to him would have belonged the honour of creating the new treatment.

Friction, with mercurial ointment, may be substituted for the Vigo plaster; and they are, in fact, quite as efficacious; but they must, as in all other cases, be performed before the eruption assumes the pustular form. An interesting case of confluent variola, which was under the care of my friend, Mr. M'Carthy, our vice-president, last year, at the Pitié, was treated by friction with mercurial ointment. I regret the want of space to give the particulars, as they would furnish me with an additional argument favourable to the cause I advocate. The application of mercury was not put in practice until the eighth day of the eruption; and though its exhibition was attended with considerable benefit, it did not prevent the suppuration; and the patient left the hospital perfectly cured, but covered with cicatrices.

It now remains for us to examine the mode of action of the metallic substance which operates such marvellous effects. Here, again, we cannot do better than call to our aid the lucid ideas of M. Briquet, who is indefatigable on this subject. In the "Archives" for Sept. 1839, we find an article devoted to the consideration of the action of mercury in variola. According to the author of this interesting memoir, (I fully adopt his views,) we are forcibly obliged to admit one of the two following explanations:—

1. The mercury acts as an antiphlogistic or resolutive, in destroying or suppressing the local inflammatory process; or

2. It exercises a specific action on the cause, whatever it be, which produces the variolous pustule.

In order to solve this interesting problem, he began by examining with care the effects of mercurial topics on the different sorts of inflammation of the skin. Artificial cutaneous phlegmasiæ were the most simple; he commenced with them. He first applied the Vigo plaster cum mercurio, which he left for twenty-four hours on the skin; and on its removal, he excited inflammation by means of cantharides, tartar-emetic ointment, frictions with croton oil, &c., and he always observed that the previous application of mercury had exercised no prophylactic influence with respect to the appearance or course of the inflammatory symptoms.

He next investigated the action of mercury on incipient inflammation, as the following experiment will elucidate.

1. Two plasters of equal dimensions, the one Vigo, the other diachylon, each covered with a little powder of cantharides, were applied to the thighs of a typhoid patient, and raised after the expiration of twenty-four hours; the vesication produced by each was perfectly identical.

2. In ten or twelve cases of chronic arthritis, frictions were made with tartar-emetic ointment, or with the croton oil; and over the inflammation caused by these applications, plasters of diachylon and Vigo were applied, *and*

*in no cases were the pustules under the mercury less violent, nor less numerous than those which were subjacent to the adhesive plaster.* M. Briquet chose the diachylon, because he had previously ascertained that this topic was quite inert in variola. The same experiments were repeated in cases of inflammations already formed, and with the same result. The inflammations to which we have been alluding, M. Briquet termed *artificial*, in contradistinction to those belonging to different pathological affections, and which may be styled *natural.* In the latter, also, it was indispensable to examine the action of mercury used topically, and here the facts become more complex; for the inflammations are variable in their course, duration, intensity, &c., and susceptible of being modified by divers circumstances. "Let us take a case of erysipelas," says M. B., "which is a malady of irregular duration; and how shall we be able to distinguish the modifications caused by mercury from those which the disease itself may experience?"

M. Ricord asserts, that he has obtained considerable benefit from the employment of the Vigo plaster in erysipelas, zona, pemphigus, exzema, acne, &c.; but I am bound to mention, in opposition to the opinion of our worthy and gifted President, that his experiments have been repeated by Messrs. Lisfranc and Velpeau, who assert, that in all cutaneous diseases to which they have applied the mercurial plaster, (variola excepted,) its exhibition has been attended with no beneficial effects. This constitutes, in my opinion, one of the strongest arguments against its *generalisation*, (permit me to use this expression.)

It furthermore appears that the external use of mercury in the *vaccine eruption*, produces effects identical with those which we have described in the variolic exanthem; namely, the *complete annihilation*, in most cases, and, *in all*, the arrest of the progress of the virus. With respect to inoculation with the pus of variola, M. Dezeimeris relates, in the journal "L'Expérience," a curious case, which is in point here, and which occurred in Lunenberg, in 1791, in the practice of Dr. Lentin. A child having been inoculated with the small pox, and accidents supervening, they were stopped by the doctor, who applied, over the eruption, a certain quantity of calomel, triturated with an emulsion of quinces.

I think it will now be admitted by all, that the mercury acts *specifically*; that it exercises a special action on the virus, whilst the latter yet exists in the *papular* state; but that it is often powerless when the pustules are developed. But, "thus far can we go, and no farther"—the following question is not, as yet, solved: "*How* does the mercury obviate the pustular eruption?"

"———adhuc sub judice lis est."

Here we must candidly admit our ignorance of the *modus operandi*; if we admit the existence of animalculæ, we may conceive that mercury kills them, as it does other parasitical beings, whose presence so incommodes the cutaneous system of men and animals; but M. Donné, whose late labours have raised microscopic observation to the rank of a science, deservedly dignified by the term *microscopie*, has not, as yet, been able to discover, in the variolic pustule, those living beings. Does the metal act as it does on blood, by diminishing its force of coagulation? Such are the problems which remain to be solved, and which afford a wide field for conjecture and research.

I have thought it necessary, gentlemen, to dwell at length on the advantages to be derived from the mercurial treatment in variola; and I shall merely add, that several cases of the disease which have fallen under my own observation, and which have been treated according to the system I have advocated before you tonight, have corroborated and strengthened my view of the question; and have convinced me that, sooner or later, mercury must form the therapeutic basis in all the varieties of variola. What objection can be urged against it? The first, and principal one, that which naturally suggests itself to the minds of all, is the danger which may result from troubling the course and progress of the eruption, by "putting it back," to use the vulgar expression. But such objections can be founded only on *theories*, and theories can be fabricated at will; and, for my part, I do not think that a single *fact* can be instanced against the many triumphant cases of radical cure, which are now ranged on our side.

In advocating the local treatment, it will, of course, be understood that I admit the propriety, nay, the necessity, of associating with it the curative indications suggested by the general state of the patient.

Let us now conclude, that the topical mercurial treatment, commenced by M. Serres, and improved by M. Briquet, is the most salutary, and offers the fairest chances of success; that it is applicable to variola, in all the diversified forms assumed by the disease; that it *never* gives rise to dangerous accidents; that, if timely and properly applied, it changes the nature of the eruption; and, finally, that it is all powerful as a preventive of cicatrices. For my own part, I look on it as almost infallible; I adopt it as my pole-star in the treatment of those cases of variola which cross my professional path.—*London Lancet.*

---

We inserted in our last number a successful case of excision of the elbow joint, and pointed out in connexion with it the importance of determining the relative mortality after excision and amputation, in cases which admit of a choice between these two methods of removing a diseased articulation. This can only be done by an analysis of a large number of observations. In order to furnish as far as possible material for such a comparison, we present to-day a successful case of

*Excision of the Head of the Humerus.*—J. M., shoemaker, married, aged 28, of sober habits, swarthy complexion, and middle stature, was admitted October 15, under the care of Mr. Liston, on account of disease of the shoulder-joint. He has always been healthy and well until the last few years, and has never had a day's illness, except what has been caused by the affection now complained of. He has for many years noticed great weakness and pain, from continued exercise, in the left arm; and, indeed, he recollects that, even when a boy, he never could use the left arm in the raised position, as in cleaning windows, without becoming unusually fatigued, and feeling an aching pain in the shoulder. He is not aware of having received any injury at this, or at any subsequent period, which could account for the symptoms; and he always considered that the weakness was "natural to him." After working at his trade, he always found his left shoulder and arm ache for some time; and though he could lift weights readily with it, or, in short, do any thing with the limb hanging by his side, he soon felt fatigue in his left shoulder whenever he was obliged to throw out his hands, as in stretching, or to work with the elbows at all raised. It is now about four years since, that he has suffered so much pain as to make him consider the matter of any consequence. At that period a dull, gnawing, aching pain began to be felt in the shoulder, extending down the arm, and often also in the bend of the elbow. At first this pain was at considerable intervals; but as he still continued his work, it recurred more and more frequently, increasing at the same time in severity, until about two years since, when he was forced to give up work altogether. At this period he says his shoulder and arm were wasted and thin, and constantly felt as if they were colder than the opposite side. There was also tenderness when he pressed his finger under the point of the shoulder, and nowhere else. He was obliged to keep his hand in a sling, and "if," as he expresses himself, "he attempted to use it, his arm fell to his side as if dead." After taking medicines and using embrocations, supplied from a dispensary which he attended for a short time, he applied to and was admitted into St. Thomas's Hospital. Here he remained for five weeks, keeping his arm quiet, and rubbing croton oil occasionally over the shoulder, so as to keep up a copious and painful eruption. He then left the hospital unrelieved, and entered St. Bartholomew's, where he remained about fifteen weeks; first under the care of a physician, who ordered cupping, leeching, and electricity, without effect; and then under Mr. Stanley, who bound his arm firmly to his side, and kept a blister open over the shoulder for several weeks. When he went out of the hospital, which he did to enter Islington workhouse, he says he was somewhat relieved. He, however, still continued to feel pain, though of a less severe character, and he could not use the limb without great suffering. He also says that when any one moved his arm about, he felt the shoulder grate as if the joint were gritty. After being in the workhouse for some time, he was directed to commence using his arm again as much as possible. The consequence was, that about eight months ago his shoulder became very painful, hot, and tender, and a swelling began to form, and gradually increased for about four months; when, having attained the size of a hen's egg, it was opened at the back of the shoulder. A large quantity of curdy matter escaped, and a similar fluid has continued to be discharged from that time to the present. The patient says that he has suffered much more pain in the limb since the abscess was opened than he did before; and of late it has extended as far as the wrist, and the back of the hand. His health has suffered from the discharge; and he has lost both strength and flesh, though his appetite continues to be pretty good. The symptoms on admission are, constant gnawing pain in the shoulder, &c., as before described, worse at night than in the day, and increased on motion, and more particularly by abduction of the limb. Tenderness over the region of the shoulder-joint and head of the humerus, and considerable loss of power in the whole extremity. Besides this, there is wasting of the arm and forearm; flatness of the shoulder, and crepitation when the head of the humerus is made to rotate against the glenoid cavity. There is a small opening on the posterior aspect of the joint, which leads to a sinus running in the direction of the head of the humerus: this Mr. Liston enlarged with a bistoury, and putting his finger into the wound, he could feel the exposed and rough bone to a considerable extent. The discharge is of a fœtid odour. The patient's bowels are open; the tongue clean; appetite pretty good; urine natural.

Oct. 20. Mr. Liston considering this a favourable case for excision of the diseased parts of bone, determined on performing the operation in a few days.

26. The patient being placed in the sitting position, with the left arm and shoulder exposed and free, Mr. Liston commenced the operation by entering his bistoury deeply beneath and behind the acromion, and cutting upon the head of the humerus vertically downwards to the extent of about three inches. From the upper angle of this incision he cut horizontally forwards, making another incision at right angles to the first, and about two inches in length. The thick, angular flap thus marked out was dissected away from the bone, and held back whilst the shoulder-joint was more freely exposed and opened from behind. The operator then taking hold of the arm, and drawing it forcibly across the chest, caused the diseased head of the humerus to be dislocated from the glenoid cavity, and appear out of the wound. The saw was at first applied to the neck of the bone, but finding that by this means an abscess in the head of it was opened, Mr. Liston made a fresh section of the shaft a little lower down, and removed the whole of the diseased parts. As the glenoid cavity felt smooth and healthy to the finger, none of the scapula was removed, and the patient was conveyed to bed, lint soaked in cold water having been first applied over the wound. Only two arteries required ligature. Six hours after the operation, all hæmorrhage having ceased, the coagula were cleared away, and the wound brought together, and retained in position by three points of interrupted suture and strips of isinglass plaster.

27. Patient passed a restless night, and is flushed and feverish this morning; pulse frequent, skin hot, tongue furred. Diffuse redness and tenderness in the neighbourhood of the wound; bowels open. He was ordered to take two tablespoonsful of the following mixture every two hours:—Three grains of extract of aconite, and six ounces of camphor mixture. Low diet. The sutures were removed, and hot fomentations frequently applied to the shoulder.

28. Less feverish; pulse still rapid, 120, though softer; heat and redness about the shoulder diminished. The patient passed a sleepless night, and is very restless and irritable: he seems to think it impossible that he should recover. To have an eighth of a grain of muriate of morphia two or three times a-day.

29. Better; pulse 96, soft; tongue clean; bowels open; slept a little last night; pain much less severe; redness about the wound very slight; fomentations continued occasionally.

31. Going on favourably; the wound is no longer red and irritable; suppuration is proceeding, and the sloughs are daily separating with the discharge; red-wash dressings; spirits better. To have a quart of beef-tea, two eggs, &c., daily.

Nov. 2. Appears pale, and rather weakened by the discharge, which is copious, rather offensive, and mixed with slough and gritty matter; perspires freely at night; pulse rather weaker; skin cool. To have a mutton-chop daily; and a draught, consisting of bark and diluted sulphuric acid, twice a-day.

6. Wound closing gradually; discharge healthy: patient takes a pint of porter daily. The arm is now kept fixed to the side in a perpendicular position, with the forearm across the chest, by means of Desault's bandage for fractured clavicle.

20. Some prominent granulations touched with sulphate of copper. Two pints of porter daily.

28. Nearly well; continue the bandage and sling; health much better; has now some colour in his cheeks, and is in good spirits.

Dec. 8. Wound has been completely closed for upwards of a week; the shoulder feels very comfortable, and free from pain; general health very good.

14. Discharged cured. The patient is now in excellent health, compared with what he has experienced for a long period back; he never suffers from any pain in the shoulder, and "feels quite another man;" he still has his arm bandaged to his side, and supported by a sling, Mr. Liston not wishing that he should be premature in trying to regain the use of the limb.—*Ibid.*

---

*Subclavian Aneurism.*—On Saturday, the 20th inst., Mr. Partridge, at the King's College Hospital, tied the subclavian artery in the first part of its course, for an aneurism which had existed in the vessel for about twelve months. After the operation, pulsation was no longer perceptible in the tumor; and for the first two days, the patient, who bore the operation very well, went on as favourably as could be expected. We believe this is the sixth time that the operation has been performed, and in no case has the individual ultimately recovered; for we regret to say that Mr. Partridge's patient died on the 24th.—*Lon. Med. Gaz.*

---

*Elixir of Vitriol.*—Among the relics of old pharmacy retained in the French Codex, is the preparation known by the names of *Teinture aromatique*, *Elixir vitriolique de Mynsicht*, and *Tinctura aromatica sulphurica.* Lest any of our young readers should confound it with the aromatic spirit of ether of the Pharm. Lond., 1824, we will observe that the Parisian elixir is made without distillation. The French recipe is as follows:

Take of the root of sweet flag, an ounce; galangale, (*Galanga officinalis,*) an ounce; chamomile flowers, half an ounce; sage leaves, half an ounce; wormwood leaves, (*Absinthium officinale,*) half an ounce; curled leave mint, (*Mentha crispa,*) half an ounce; cloves, three drachms; cinnamon, three drachms; cubebs, three drachms; nutmegs, three drachms; ginger, three drachms; aloes wood (*Aloexylum agallochum,*) one drachm; lemon peel, one drachm; white sugar, three ounces; alcohol, two pounds; sulphuric acid, four ounces.

The solid substances are to be reduced to a coarse powder, then put into a matras, and eight ounces of alcohol poured over them. After forty-eight hours' maceration, the sulphuric acid is to be gradually mixed with them, and the mixture is to stand for twenty-four hours; the rest of the alcohol is then to be added.—*Ibid.*

### METEOROLOGICAL REGISTER FOR APRIL, 1841.

*Kept at the Pennsylvania Hospital, by J. Conrad.*

| Date. | Thermometer. Max. | Min. | 9 AM. | Barometer. 10 AM | 3 PM. | Dew Point | Winds. Direction. | Force | Rain. | REMARKS. |
|---|---|---|---|---|---|---|---|---|---|---|
| 1 | 59 | 38 | 47 | 29.94 | 29.92 | 42 | SW. | 2 | .033 | Light rain at 2½ and 8½ AM; morning cloudy; aft'n clear. |
| 2 | 69 | 42 | 58 | 29.89 | 29.68 | 41 | SW.NW. | 1-6 | .190 | Morn fair; aft'n cloudy; thunder gust at 6 PM; gale at night. |
| 3 | 51 | 36 | 42 | 30.19 | 30.18 | 20 | NW.SW. | 1 | | Clear. |
| 4 | 52 | 36 | 48 | 30.20 | 30.00 | 43 | SE. | 2 | .997 | Morning cloudy; rain aft'n and ev'g. |
| 5 | 59 | 46 | 50 | 29.90 | 29.90 | 38 | NW.W. | 2 | .022 | Clear; cloudy from 4½ PM; rain in evening. |
| 6 | 57 | 41 | 49 | 30.16 | 30.16 | 34 | NW. | 1 | .020 | Light rain at 6 AM; clear from 10 AM. |
| 7 | 60 | 41 | 47 | 30.18 | 29.97 | 38 | SE.NE. | 2 | | Morning cloudy; afternoon and evening partly clear. |
| 8 | 56 | 42 | 45 | 29.90 | 29.90 | 39 | NW.W. | 1 | .008 | Light rain at 7 AM; clear from 10½ AM. |
| 9 | 68 | 42 | 56 | 29.89 | 29.66 | 50 | SW. | 3 | | Clear. |
| 10 | 56 | 31 | 47 | 29.90 | 29.88 | 28 | NE. | 3 | | Morn cloudy; snow from 2 to 9½ PM; therm 36° at 3 PM. |
| 11 | 44 | 30 | 33 | 30.23 | 30.23 | 20 | NE. | 1 | | Clear; evening cloudy. |
| 12 | 35 | 32 | 34 | 30.15 | 30.05 | 30 | NE. | 4 | 1.150 | Snow storm from 6 AM. all day; 9 in. deep at 9 PM. |
| 13 | 37 | 31 | 39 | 30.00 | 29.96 | 30 | NW. | 1 | | Clear from 8 AM. |
| 14 | 49 | 33 | 41 | 30.00 | 30.07 | 24 | NE.NW. | 2 | 1.032 | Rain till 4 AM; snow from 4 to 7½ AM; clear from 10 AM. |
| 15 | 48 | 30 | 38 | 30.47 | 30.44 | 19 | NW. | 1 | | Very clear. |
| 16 | 58 | 31 | 49 | 30.53 | 30.43 | 31 | SW. | 1 | | Clear. |
| 17 | 61 | 41 | 51 | 30.15 | 30.01 | 47 | SW. | 1 | .012 | Light rain early in morning; cloudy. |
| 18 | 66 | 40 | 63 | 29.68 | 29.79 | 39 | SW.NW. | 4 | .020 | Light rain in night; clear from 9½ AM. |
| 19 | 56 | 36 | 43 | 30.21 | 30.11 | 27 | NW.SW. | 2 | | Cloudless. |
| 20 | 56 | 41 | 56 | 30.13 | 30.10 | 41 | SW.SE. | 2 | .344 | Cloudy; rain from 11 AM. all day. |
| 21 | 58 | 43 | 54 | 29.96 | 30.00 | 40 | NE.N. | 3 | | Light rain till 8 AM; cloudy all day. |
| 22 | 51 | 41 | 48 | 30.13 | 30.06 | 41 | NE. | 3 | .384 | Cloudy; rain in aft. and ev'g. |
| 23 | 58 | 45 | 53 | 30.01 | 30.02 | 49 | NE. | 3 | | Rain till 8 AM; cloudy. |
| 24 | 62 | 49 | 54 | 30.12 | 30.07 | 52 | NE. | 1 | | Cloudy. |
| 25 | 63 | 53 | 57 | 30.11 | 30.10 | 52 | NE. | 1 | | Cloudy. |
| 26 | 65 | 50 | 52 | 30.00 | 29.85 | 51 | NE.SW. | 1 | | Morning cloudy; aft'n clear. |
| 27 | 59 | 44 | 56 | 29.74 | 29.80 | 40 | NW. | 3 | .356 | Rain till 9 AM; aft'n clear. |
| 28 | 62 | 41 | 52 | 30.09 | 30.00 | 32 | NW. | 1 | | Fair, but hazy. |
| 29 | 49 | 43 | 49 | 29.80 | 29.50 | 45 | SE.NE. | 3 | 1.500 | Rain from 8½ AM till 7 PM. |
| 30 | 53 | 45 | 52 | 29.15 | 29.20 | 40 | NW. | 3 | .050 | Rain from 10 to 12 AM, and 2 to 6 PM; evening clear. |
| Mean | 56.23 | 39.80 | 48.76 | 30.02 | 29.96 | 37.39 | | | 6.456 | |

| | | | |
|---|---|---|---|
| Mean temperature, | 48° | Clear days, - - | 13½ |
| " pressure, | 29.99 inches. | Clouds, rain, &c. - | 16½ |
| " dew-point, | 37.39° | But 4 entirely clear days. | |

*Winds*—W. to S. 8½ days; S. to E. 4 days; E. to N. 10 days; N. to W. 7½ days.

